# LIGHT controls distinct homeostatic and inflammatory gene expression profiles in esophageal fibroblasts via differential HVEM and LTβR-mediated mechanisms

**DOI:** 10.1038/s41385-021-00472-w

**Published:** 2021-12-13

**Authors:** Mario C. Manresa, Amanda Wu, Quan M. Nhu, Austin W. T. Chiang, Kevin Okamoto, Haruka Miki, Richard Kurten, Elaine Pham, Loan D. Duong, Nathan E. Lewis, Praveen Akuthota, Michael Croft, Seema S. Aceves

**Affiliations:** 1 Department of Pediatrics, University of California, San Diego, CA USA; 2 Division of Allergy Immunology, San Diego, CA USA; 3 La Jolla Institute for Immunology, La Jolla, CA USA; 4 Division of Gastroenterology and Hepatology, Scripps Clinic, San Diego, CA USA; 5 Department of Physiology and Biophysics, University of Arkansas for Medical Sciences, Arkansas Children’s Hospital Research Institute, Little Rock, AR USA; 6 Division of Pulmonary, Critical Care, and Sleep Medicine, University of California San Diego, La Jolla, CA USA; 7 Department of Medicine, University of California, San Diego, CA USA; 8 Rady Children’s Hospital San Diego, San Diego, CA USA

## Abstract

Fibroblasts mediate tissue remodeling in eosinophilic esophagitis (EoE), a chronic allergen-driven inflammatory pathology. Diverse fibroblast subtypes with homeostasis-regulating or inflammatory profiles have been recognized in various tissues, but which mediators induce these alternate differentiation states remain largely unknown. We recently identified that TNFSF14/LIGHT promotes an inflammatory esophageal fibroblast in vitro. Herein we used esophageal biopsies and primary fibroblasts to investigate the role of the LIGHT receptors, herpes virus entry mediator (HVEM) and lymphotoxin-beta receptor (LTβR), and their downstream activated pathways, in EoE. In addition to promoting inflammatory gene expression, LIGHT down-regulated homeostatic factors including WNTs, BMPs and type 3 semaphorins. In vivo, WNT2B^+^ fibroblasts were decreased while ICAM-1^+^ and IL-34^+^ fibroblasts were expanded in EoE, suggesting that a LIGHT-driven gene signature was imprinted in EoE versus normal esophageal fibroblasts. HVEM and LTβR overexpression and deficiency experiments demonstrated that HVEM regulates a limited subset of LIGHT targets, whereas LTβR controls all transcriptional effects. Pharmacologic blockade of the non-canonical NIK/p100/p52-mediated NF-κB pathway potently silenced LIGHT’s transcriptional effects, with a lesser role found for p65 canonical NF-κB. Collectively, our results show that LIGHT promotes differentiation of esophageal fibroblasts toward an inflammatory phenotype and represses homeostatic gene expression via a LTβR-NIK-p52 NF-κB dominant pathway.

## Abstract (web summary)

▓

## Graphical abstract

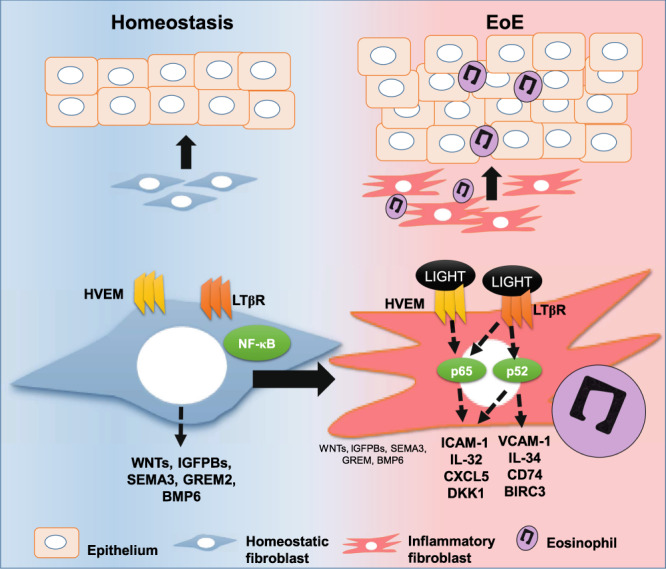

## Introduction

Fibroblasts are increasingly being recognized as plastic cells that can acquire diverse phenotypes in homeostasis and disease^1–4^. Until recently, these cells were thought mainly to participate in tissue healing and, upon activation during disease states, to acquire a myofibroblast phenotype and deposit excess extracellular matrix (ECM) thereby leading to fibrosis and wound contracture^5,6^. However, recent single cell studies in Th2 and Th1 diseases such as eczema, inflammatory bowel disease, and rheumatoid arthritis have documented the existence of unique fibroblast subsets with distinct transcriptional profiles that allow them to produce homeostatic or inflammatory factors^1,2,4,7^. This is well documented in the intestinal tract, where fibroblast populations localized around colonic crypts produce WNT factors, type 3 semaphorins (SEMA3), or bone morphogenetic proteins (BMPs) to sustain epithelial renewal^8,9^. Similarly, a stromal population expressing the WNT agonist R-spondin3 is thought critical for intestinal epithelial recovery after injury^10^. In ulcerative colitis, these epithelial homeostasis-regulating fibroblasts are diminished, and fibroblasts that express inflammatory mediators emerge^4^. In addition, multicellular inflammatory modules that are rich in inflammatory fibroblasts can predict resistance to TNF-blocking therapies in Crohn’s disease^2^. Considering this cumulative evidence of the critical roles of fibroblasts in homeostasis and inflammation, elucidating the factors that drive fibroblast functional changes may represent a new avenue to identify targets for the treatment of chronic inflammatory diseases leading to tissue fibrosis.

Eosinophilic esophagitis (EoE) is an allergic disease of the esophagus defined by a robust and eosinophil-predominant inflammation that causes clinical symptoms of chest pain, failure to thrive and dysphagia. A significant subset of EoE patients present with persistent eosinophilic inflammation that does not respond to the currently available therapies of elimination diets and topical corticosteroids^11–13^. Patients who are therapy resistant or untreated almost uniformly progress from an inflammatory to a fibrotic disease that leads to esophageal narrowing and lost luminal patency. Pro-fibrotic factors such as TGF-β1 are increased in the EoE esophagus and TGF-β1-driven fibroblasts have higher collagen expression. It is thought that tissue fibroblasts, myofibroblasts, and canonical TGF-β1 signals are pivotal in the ECM changes in EoE that mediate esophageal strictures^14–16^. However, the existence of other fibroblast subtypes in EoE, including pro-inflammatory fibroblasts, and the identity of the stimuli that promote alternate fibroblast differentiation in the esophagus is not well understood.

We previously showed that a member of the tumor necrosis factor superfamily (TNFSF) of cytokines termed TNFSF14/LIGHT is produced in the esophagus during active EoE^17^. In vitro, LIGHT imposes an inflammatory phenotype on fibroblasts that closely resembles that described in the colon, expressing common markers such as ICAM-1, IL-32, IL-33 or CD74, among others^4,17^. LIGHT has gained momentum as a mediator of tissue remodeling that also induces inflammatory gene expression in lung fibroblasts and epithelial cells^18–20^. LIGHT signals through two receptors termed herpes virus entry mediator (HVEM) and lymphotoxin-beta-receptor (LTβR) and the contribution of each receptor to the LIGHT transcriptome can be cell and context dependent^21–27^.

Here, we investigated the effect of LIGHT on homeostatic and inflammatory gene expression, and the involvement of HVEM and LTβR in LIGHT-mediated responses, in esophageal fibroblasts. We elucidated the contribution of canonical and non-canonical NF-κB pathways to LIGHT-driven responses and defined the presence of LIGHT-regulated mediators in the active EoE esophagus. Collectively, our results reveal unique expression profiles and functions of both LIGHT receptors and a key role of the non-canonical NIK/p100/p52 NF-κB pathway in LIGHT-mediated fibroblast differentiation.

## Results

### HVEM and LTβR have distinct contributions to LIGHT-modulated gene expression in esophageal fibroblasts

**Fig. 1:**
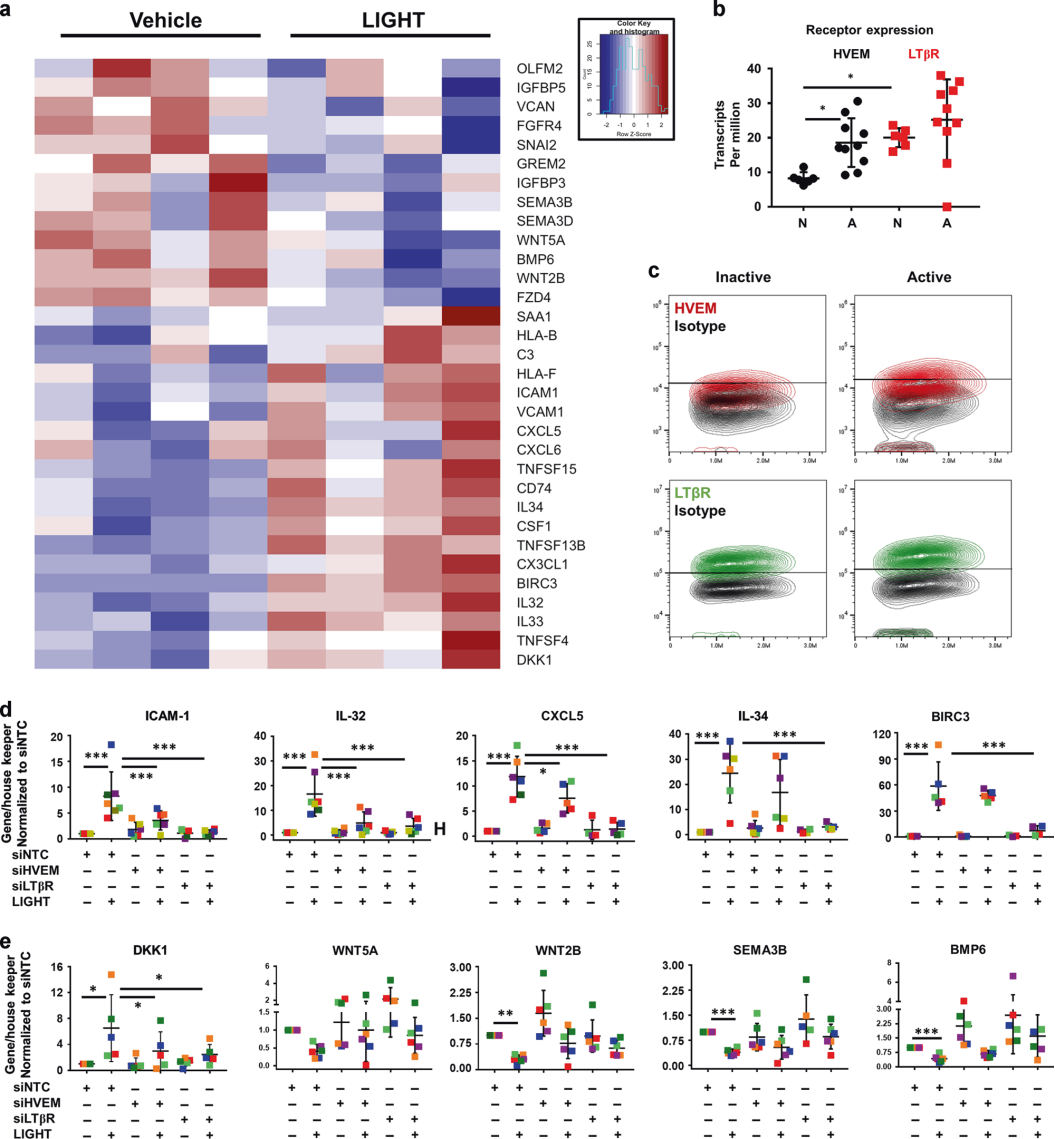
HVEM and LTβR contribute to LIGHT-mediated responses in esophageal fibroblasts. **a** Heat map of representative homeostatic and inflammatory mediators comparing vehicle or LIGHT treated esophageal fibroblasts from 4 different donors (>1.5 fold, *p* < 0.05). **b** Comparison of HVEM and LTβR expression (TPM) in normal esophagus (*n* = 6) and active EoE biopsies (*n* = 10) from Sherrill et al. 2014. **c** Representative plots showing the frequency of HVEM (red) and LTβR (green)-expressing cells compared to isotype control in active or inactive EoE fibroblasts (*n* = 6). qRT-PCR expression of inflammatory (**d**) or homeostatic (**e**) genes in cells transfected with non-targeting RNA (siNTC) or siRNAs against HVEM (siHVEM) or LTβR (siLTβR) in vehicle or LIGHT-treated (24 h) cells (each colored dot represents fibroblasts from a different donor, *n* ≥ 5). *p < 0.05 and ***p < 0.001.

We have previously reported that LIGHT up-regulates a distinct subset of genes with inflammatory properties in esophageal fibroblasts (Fig. 1a)^17^. In addition to its ability to up-regulate inflammatory mediators, an analysis of the LIGHT-repressed transcriptome revealed that LIGHT down-regulates multiple mediators of homeostatic functions including WNT factors (WNT5A, WNT2B), WNT receptors and targets (FZD4 and OLFM2), bone morphogenetic proteins (BMP6) or type 3 semaphorins (SEMA3, Fig. 1a). Interestingly, many of these factors contribute to the maintenance of epithelial homeostasis and regeneration in digestive tissues including colon and esophagus^8,28,29^. In line with this, an Ingenuity Pathway Analysis linked the LIGHT down-regulated transcriptome to processes such as cellular and tissue growth or development, epithelial to mesenchymal transition and cell-to-cell communication (Supplementary Fig. 1). Therefore, in addition to the activation of inflammatory responses, LIGHT represses gene expression programs that may participate in the maintenance of epithelial homeostasis.

LTβR is stably expressed in esophageal fibroblasts from different backgrounds, whereas HVEM expression can be increased by TGF-β1^17^. In line with this, an analysis of the transcript expression of these receptors in a previously published data set comparing normal esophagus to active EoE biopsies demonstrated that LTβR was more highly expressed than HVEM in normal biopsies (Fig. 1b)^30^. Moreover, HVEM was significantly up-regulated in active EoE, whereas LTβR expression was not different between EoE and normal biopsies (Fig. 1b). The relative level of expression of surface HVEM was generally increased in fibroblasts from active EoE patients compared to patients in remission (inactive EoE), but was lower than the level of LTβR, which was stable in either disease state (Fig. 1c and Supplementary Fig. 2A). To analyze the contribution of these receptors to the LIGHT transcriptome, we used siRNAs to knockdown either receptor in esophageal fibroblasts from different normal donors (Supplementary Fig. 2B-C). LTβR silencing abrogated the induced expression of all of the LIGHT-regulated transcripts that we analyzed whereas knockdown of HVEM produced more selective effects. ICAM-1, IL-32 and CXCL5 were up-regulated in response to LIGHT in the presence of non-targeting control RNA (siNTC) and knockdown of either HVEM or LTβR prevented their LIGHT-mediated up-regulation. In contrast, IL-34 or BIRC3 were only affected by a loss of LTβR (Fig. 1d). Transcripts for DKK1, an inhibitor of WNT signaling, were up-regulated by LIGHT and this was blocked by siHVEM or siLTβR (Fig. 1e). Moreover, the basal expression of WNT5A, WNT2B, SEMA3B and BMP6 was repressed by LIGHT and partially restored by a loss of either receptor (Fig. 1e). Together, our results show that both receptors play a role in the inflammatory-promoting and homeostasis-repressing gene expression profiles driven by LIGHT in esophageal fibroblasts. However, our data suggests that LTβR has a dominant role, as it is stably expressed regardless of disease state and required for the modulation of all genes tested.

**Fig. 2:**
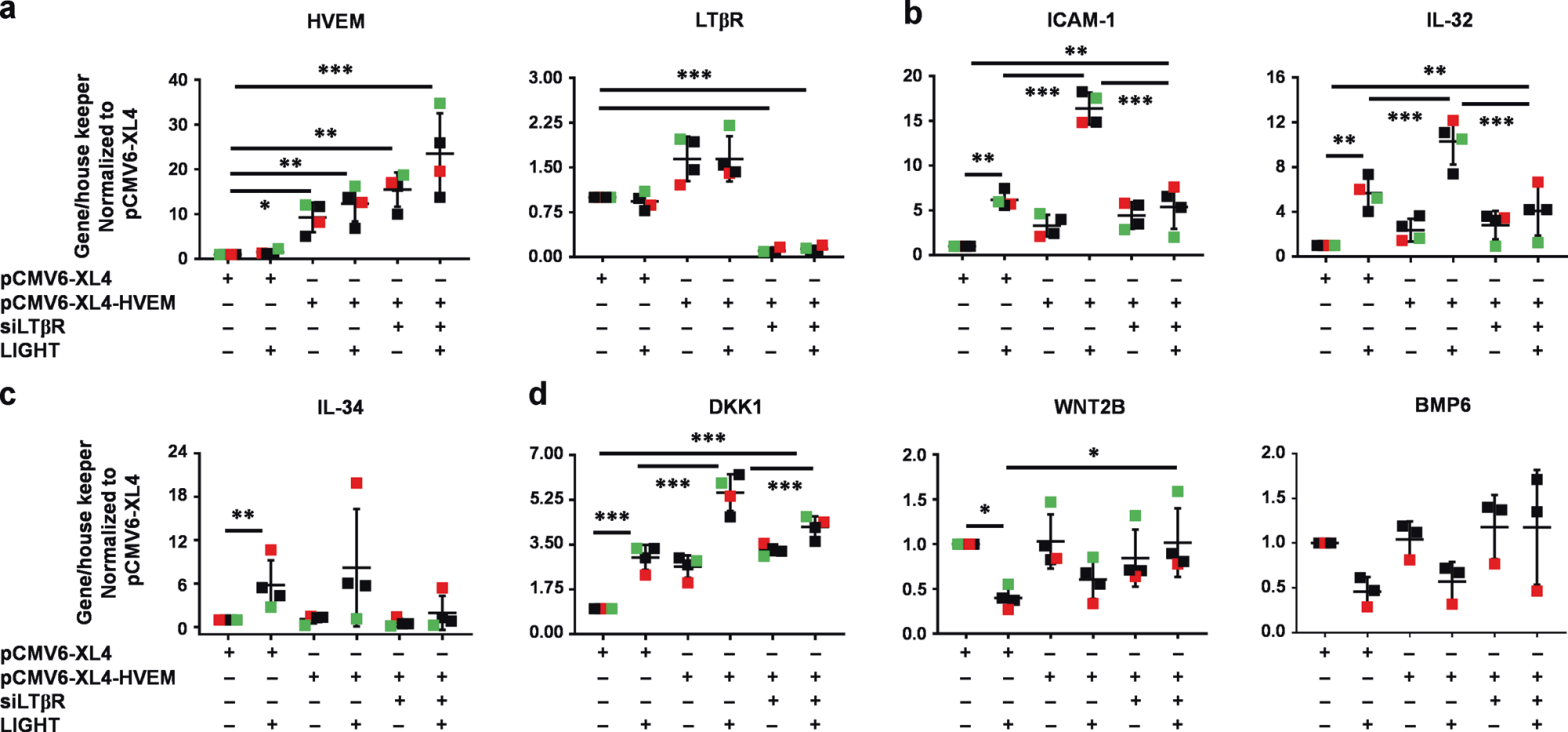
Overexpression of HVEM partially modulates LIGHT-mediated gene expression in esophageal fibroblasts. qRT-PCR of: HVEM and LTβR (**a**); ICAM-1 and IL-32 (**b**); IL-34 (**c**); and DKK1, WNT2B and BMP6 (**d**), in normal esophageal fibroblasts transfected with empty vector (pCMV6-XL4), HVEM-OE (pCMV6-XL4-HVEM) or a combination of HVEM-OE + siLTβR, and untreated or treated with LIGHT (*n* ≥ 3). Each colored dot represents fibroblasts from a different donor, *p < 0.05, **p < 0.01 and ***p < 0.001.

TGF-β1 enhances HVEM expression on esophageal fibroblasts, and cells pretreated with TGF-β1 and stimulated with LIGHT display enhanced expression of genes such as IL-32 or ICAM-1^17^ corresponding to the requirement for HVEM for modulating these molecules shown above. To further assess the isolated importance of HVEM, we overexpressed HVEM (HVEM-OE) in the presence of LTβR silencing (Fig. 2a and Supplementary Fig. 2D). HVEM overexpression significantly enhanced LIGHT-mediated ICAM-1 and IL-32 transcripts compared to cells transfected with empty vector, but this was still blocked by siLTβR (Fig. 2b). LIGHT-mediated IL-34 was not enhanced by HVEM overexpression and correspondingly was inhibited by siLTβR (Fig. 2c). Increased HVEM significantly induced the expression of the WNT inhibitor DKK1 even in the absence of LIGHT and this was further enhanced by LIGHT stimulation dependent on LTβR. This may indicate that HVEM regulates DKK1 by both LIGHT-dependent and independent mechanisms. In contrast, WNT2B and BMP6 downregulation by LIGHT was not affected by HVEM-OE and only dependent on LTβR knock-down (Fig. 2d). Therefore, HVEM modulation likely reflects a mechanism whereby esophageal fibroblasts fine tune the expression of specific subsets of LIGHT-LTβR-regulated targets.

### HVEM and LTβR-dependent mechanisms contribute to fibroblast-eosinophil tethering in co-culture

**Fig. 3:**
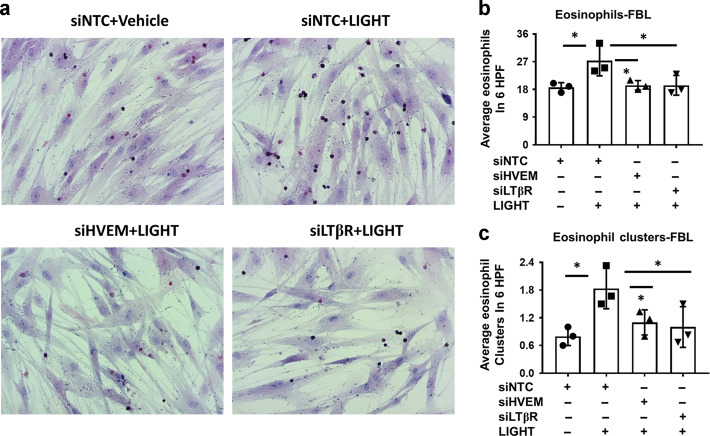
HVEM and LTβR-mediated mechanisms contribute to fibroblast-eosinophil tethering in co-culture. **a** Representative haematoxylin/eosin stained co-cultures of fibroblasts and eosinophils in fibroblasts transfected with non-targeting control RNA (siNTC) or siRNAs against HVEM (siHVEM) or LTβR (siLTβR) in vehicle or LIGHT treated (24 h) cells followed by co-culture with eosinophils for 8 h in the absence of LIGHT (n = 3). Quantification of total eosinophils (**b**) or eosinophil clusters (**c**) in cells treated as in **a** (each dot represents fibroblasts from an independent donor). *p < 0.05.

In previous studies we showed that eosinophils tether to fibroblasts in co-culture, that this is enhanced by LIGHT pre-treatment of fibroblasts, and requires ICAM-1^17^. In concordance with the data shown above on the requirement for HVEM and LTβR for upregulating ICAM-1, transfection of fibroblasts with siHVEM or siLTβR led to reduced eosinophil tethering to LIGHT-treated fibroblasts and blocked the formation of multi-eosinophil clusters on the surface on fibroblasts (Fig. 3a–c). These data show functional contributions of both LIGHT receptors to one LIGHT-mediated inflammatory function in esophageal fibroblasts.

### Spatial analysis of LIGHT-regulated homeostatic and inflammatory genes reveals imbalanced fibroblasts populations in EoE

**Fig. 4:**
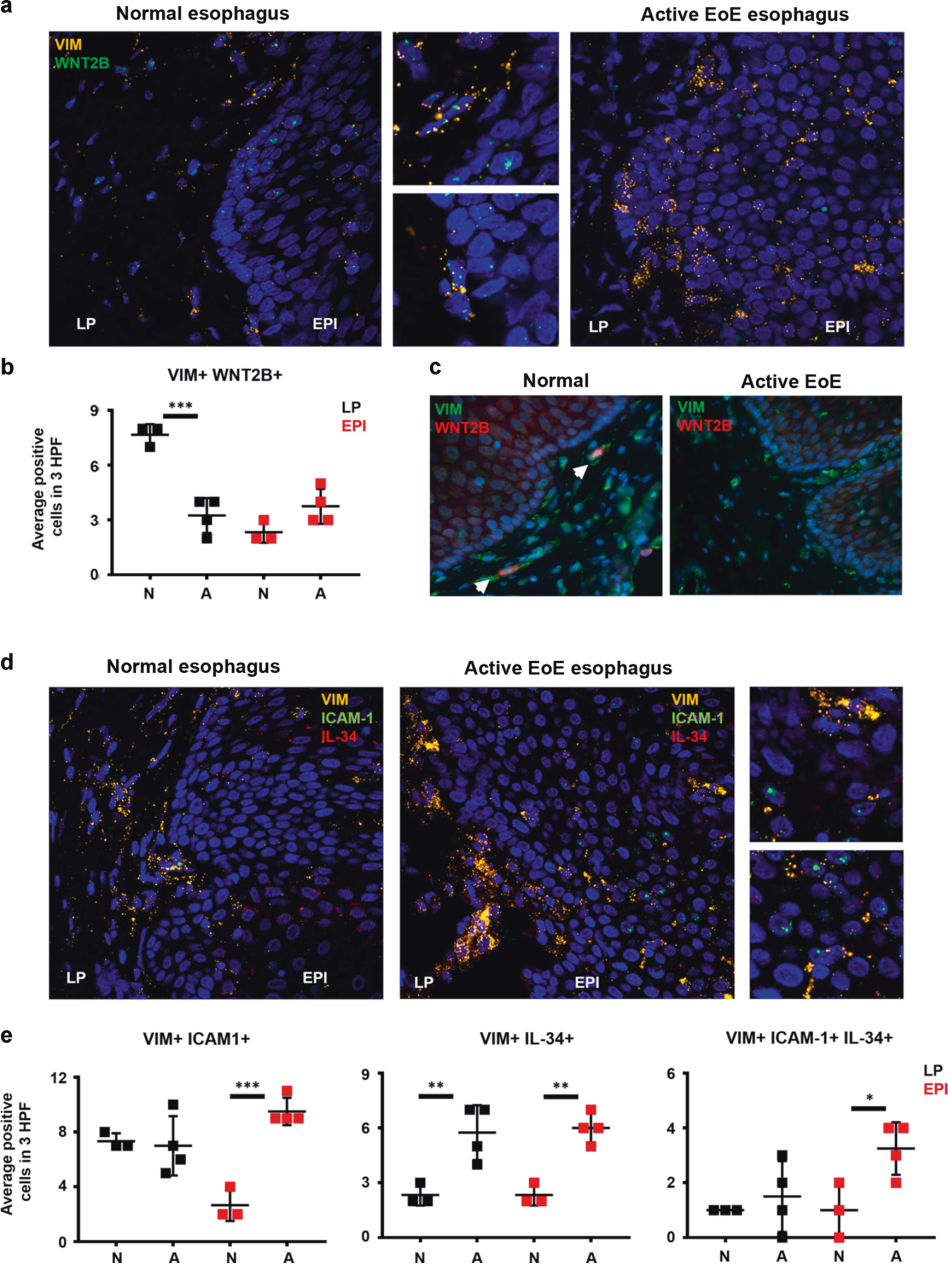
Localization of VIM + cells expressing homeostatic and inflammatory markers reveals functional changes in EoE. **a** Representative images of normal (*n* = 3) and active EoE esophagus (*n* = 4) hybridized with specific probes for VIM (yellow) and WNT2B (green, LP = lamina propria, EPI = epithelium). **b** Immunofluorescence staining of normal (*n* = 3) and active EoE esophagus (*n* = 4) for VIM (green) and WNT2B (red). **c** Quantification of VIM+ WNT2B+ cells in LP and EPI comparing normal and active EoE esophagus. **d** Representative images of normal (*n* = 3) and active EoE esophagus (*n* = 4) hybridized with specific probes for VIM (yellow), ICAM-1 (green) and IL-34 (red). **e** Quantification of VIM+ ICAM-1+ , VIM+ IL-34+ and VIM+ ICAM-1+ IL-34+ cells in LP and EPI comparing normal and active EoE esophagus. White arrows in c point positive cells. *p < 0.05, **p < 0.01 and ***p < 0.001.

Substantive literature supports the existence of fibroblasts with inflammatory and homeostatic or reparative profiles in various tissues, and our data suggested that LIGHT could be one molecule that promotes an inflammatory phenotype while down-regulating homeostatic factors^1,4,8,31^. To test whether signatures of LIGHT-regulated homeostatic and inflammatory fibroblasts are present in EoE, we performed RNA in situ hybridization (RNAscope) in normal esophagus and active EoE biopsies. We previously reported that vimentin (VIM) and CD90 can be used to identify fibroblasts in the esophagus^17^. A preliminary analysis revealed that VIM transcripts are more abundantly expressed than CD90 suggesting that this may be a more sensitive marker to identify fibroblasts by RNAscope (not shown). Using VIM as a common fibroblast marker and WNT2B as a marker of a fibroblasts with homeostatic profile, we found abundant VIM^+^WNT2B^+^ cells in the lamina propria (LP) of the normal esophagus (Fig. 4a). This was also confirmed by immunofluorescence and by quantification of cells concomitantly expressing VIM and WNT2B transcripts (Fig. 4b, c). In contrast, in active EoE, the number of VIM^+^WNT2B^+^ cells in the LP was significantly lower indicating a potential loss of homeostatic fibroblasts or a transition of this fibroblast to a pathogenic state (Fig. 4a–c). To understand whether fibroblasts with inflammatory profiles emerge in EoE disease, we analyzed the expression of ICAM-1 and IL-34 based on our data in Fig. 1. This analysis showed that VIM^+^ICAM-1^+^ cells were similarly abundant in the LP in the normal and EoE esophagus, but that the number of VIM^+^ICAM-1^+^ cells detected in the epithelial compartment (EPI) was significantly upregulated in EoE (Fig. 4d and e). VIM^+^IL-34^+^ cells were also upregulated in active EoE in both LP and EPI compartments and VIM^+^ICAM-1^+^IL-34^+^ cells were highest in the active EoE EPI (Fig. 4d, e). These data suggest that a shift in fibroblasts function may contribute to disease, and support an action of LIGHT on fibroblast differentiation in the esophagus, consistent with our prior finding of abundant LIGHT^+^ cells in active EoE^17^.

### LIGHT-modulated inflammatory and homeostatic functions in esophageal fibroblasts are imprinted in EoE

**Fig. 5:**
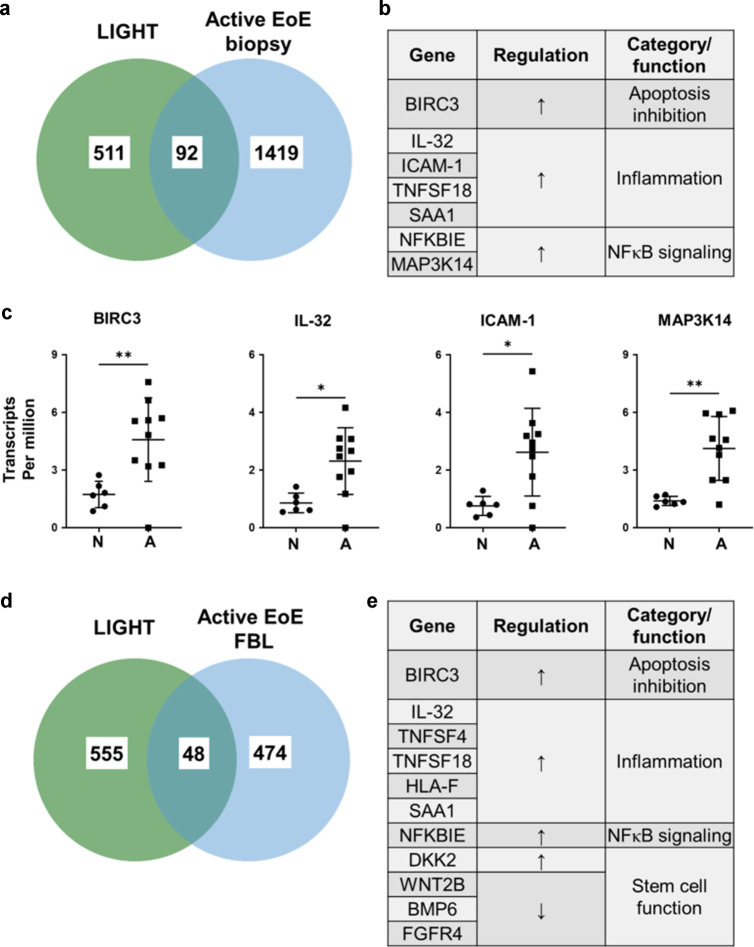
Comparison of LIGHT-mediated and basal EoE transcriptomes reveals common regulated gene signatures. Venn diagram (**a**) and list of selected commonly regulated genes (**b**) comparing genes differentially expressed in LIGHT-treated normal esophageal fibroblasts compared to vehicle (*n* = 4) and active EoE biopsies (*n* = 10) compared to normal esophagus (*n* = 6). **c** Normalized transcript per million analyses of BIRC3, IL-32, ICAM-1 and MAP3K14 in normal and active EoE esophageal biopsies. Venn diagram (**d**) and list of selected commonly regulated genes (**e**) comparing genes differentially expressed in LIGHT-treated normal esophageal fibroblasts compared to vehicle (*n* = 4) and basal expression in active EoE fibroblasts compared to normal (*n* = 4).

The global transcriptome dysregulated in esophageal biopsies from active EoE patients compared to normal esophagus has previously been published^30^. To further understand whether the phenotypic changes induced by LIGHT in esophageal fibroblasts are present in EoE, we analyzed whether the LIGHT-driven fibroblast transcriptome aligned with the global EoE transcriptome. This analysis revealed a total of 92 common differentially expressed genes, including up-regulated genes such as IL-32, ICAM-1, BIRC3, CXCL5 and SAA1 (Fig. 5a–c). Our analysis of common up-regulated genes also revealed enhanced expression of MAP3K14 (NF-κB-inducing kinase or NIK), which is a key component of the non-canonical NF-κB pathway (Fig. 5a–c). To further substantiate the hypothesis that LIGHT may drive fibroblast differentiation in EoE, an RNA sequencing analysis of the basal transcriptome present in fibroblasts from patients with active EoE isolated and cultured in vitro was performed compared to fibroblasts from the normal esophagus cultured under the same conditions. This revealed differential expression of 522 genes, including 187 upregulated and 335 downregulated targets (Fig. 5d). Comparison between the EoE fibroblast transcriptome with that induced by LIGHT in normal esophageal fibroblasts revealed 48 common differentially expressed genes (DEGs). Among these, 19 were upregulated by LIGHT and in EoE fibroblasts, 15 were down-regulated by LIGHT and in EoE fibroblasts, and 14 were regulated in opposing directions (Fig. 5d–e). Among the inflammatory targets, we found some of the top LIGHT-regulated markers (IL-32 and BIRC3), inflammatory and acute phase molecules (TNFSF4, TNFSF18, HLA-F and SAA1) as well as NF-κB signaling molecules and WNT inhibitors (NFKBIE and DKK2, Fig. 5d–e). Commonly repressed genes included WNT2B and BMP6 (Figure D-E). This data indicates that signatures of LIGHT-driven responses are conserved in fibroblasts from active EoE biopsies in culture and identifies potential fibroblast phenotype markers and functions in EoE.

### LIGHT induces kinetically different canonical and non-canonical NF-κB pathways in esophageal fibroblasts

**Fig. 6:**
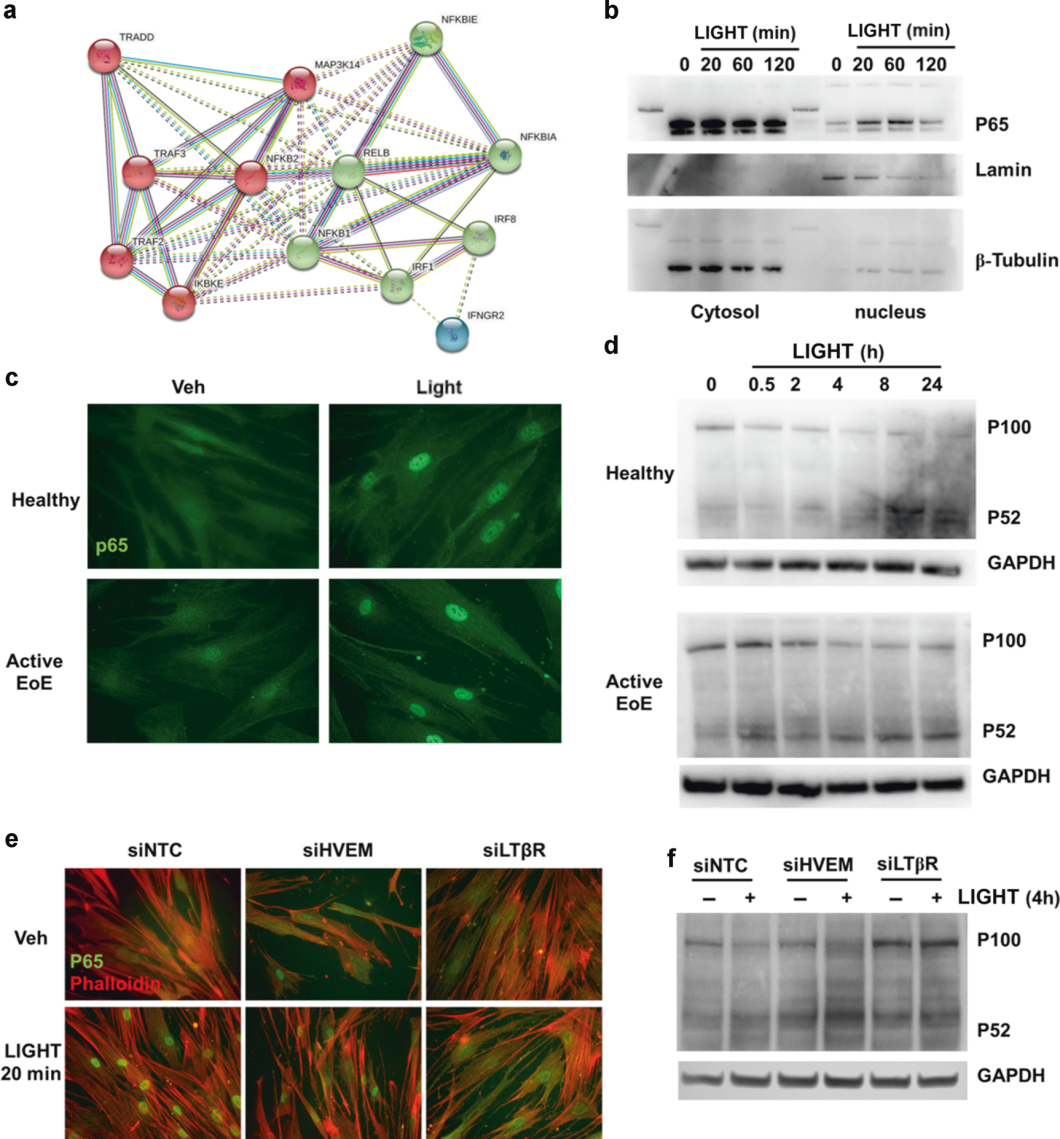
LIGHT activates canonical and alternative NF-κB signaling pathways in esophageal fibroblasts. **a** STRING analysis of molecular signaling-related genes up-regulated by LIGHT in normal esophageal fibroblasts (>1.5 fold, *p* < 0.05). **b** Representative western blot (WB) comparing p65 translocation in nuclear vs cytosolic extracts from normal esophageal fibroblasts treated with LIGHT for the indicated times (*n* = 3). **c** Representative images of normal or active EoE esophageal fibroblast monolayers stained for p65 after 20 min of LIGHT stimulation (*n* = 3). **d** Representative WB comparing p100 cleavage into p52 in whole cell lysates from normal or active EoE esophageal fibroblasts treated with LIGHT for the indicated times (*n* = 3). Representative images of p65 translocation (**e**) and representative western blot of p100 cleavage (**f**) in cells transfected with scramble RNA (siNTC) or siRNAs against HVEM (siHVEM) or LTβR (siLTβR) and untreated or treated with LIGHT for the times indicated (*n* = 3).

A STRING^32^ analysis of signaling mediators upregulated by LIGHT at the transcript level in esophageal fibroblasts showed that 3 central components of the non-canonical NF-κB pathway (NFKB2/p100, MAP3K14/NIK and RELB) and one canonical pathway component (NFKB1/p50) are at the center of the predicted LIGHT-activated signaling interactions (Fig. 6a). Time course immunoblotting experiments in fibroblasts validated the canonical p65/RelA pathway with nuclear translocation and subsequent decay after 20 min and 2 h of LIGHT stimulation, respectively (Fig. 6b). Immunofluorescence staining for p65 confirmed its nuclear accumulation after 20 min in normal, as well as active EoE esophageal fibroblasts (Fig. 6c). Furthermore, cleavage of p100 into p52, an event triggered by the activation of a NIK (MAP3K14)-dependent non-canonical NF-κB response, was found starting at 4 h in normal esophageal fibroblasts and 2 h in active EoE fibroblasts following LIGHT treatment which was sustained for up to 24 h (Fig. 6d). To understand the receptor dependence of each specific pathway, we analyzed early p65 translocation and p100 cleavage in cells transfected with siNTC, siHVEM or siLTβR. Knock-down of HVEM or LTβR reduced p65 nuclear accumulation (Fig. 6e). In contrast, knockdown of LTβR but not HVEM, suppressed the cleavage of p100 into p52 (Fig. 6f). These data show that LIGHT activates both canonical and non-canonical NF-κB pathways in esophageal fibroblasts and, consistent with its broader effects on gene transcription, that LTβR regulates both pathways, while HVEM only regulates p65-mediated signaling.

### Inhibition of the non-canonical NF-κB pathway has a dominant suppressive effect on LIGHT-mediated responses in esophageal fibroblasts

**Fig. 7:**
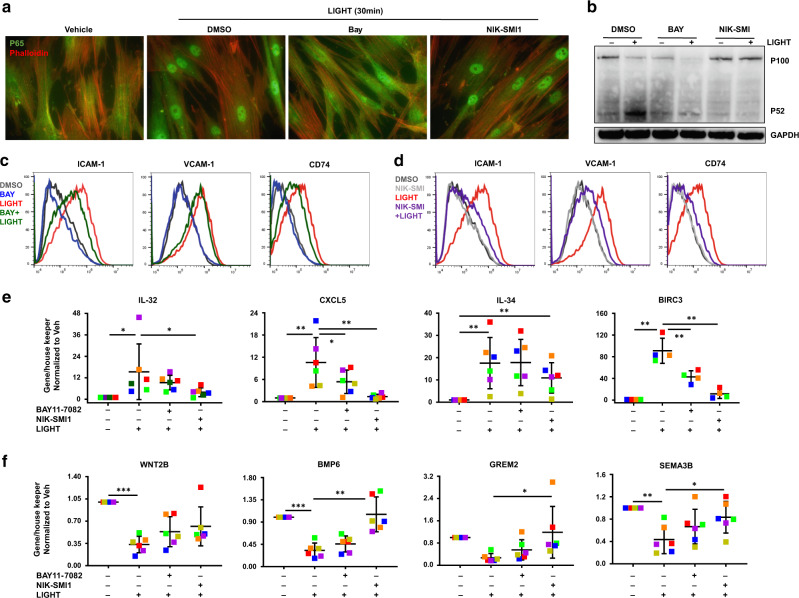
NIK inhibition has a dominant suppressive role on LIGHT responses in esophageal fibroblasts. Representative images of p65 translocation (**a**) and representative western blot of p100 cleavage (**b**) in cells pre-treated with BAY11-7082 or NIK-SMI1 for 1 h and then treated with LIGHT for the times indicated (*n* = 3). Representative histograms of flow cytometry of ICAM-1, VCAM-1 and CD74 in normal esophageal fibroblasts pre-treated with BAY11-7082 (**c**) or NIK-SMI1 (**d**) for 1 h and then treated with LIGHT for 24 h (*n* = 3). RT-PCR of inflammatory (**e**) and homeostatic (**f**) genes in esophageal fibroblasts pre-treated with BAY11-7082 or NIK-SMI1 for 1 h and then treated with LIGHT for 24 h (*n* = 6). Each colored dot represents fibroblasts from an independent donor. *p < 0.05, **p < 0.01 and ***p < 0.001.

To define the role of these NF-κB pathways in the response to LIGHT, we used pharmacological inhibitors. The canonical pathway inhibitor, BAY11-7082, repressed LIGHT-mediated translocation of p65 into the nucleus but did not prevent the cleavage of p100. In contrast, the NIK inhibitor NIK-SMI1 did not affect p65 nuclear accumulation but blocked p100 cleavage in normal esophageal fibroblasts (Fig. 7a, b). At 24 h post-LIGHT stimulation, p65 inhibition had mild inhibitory effects on LIGHT-mediated ICAM-1 and CD74 up-regulation and did not prevent VCAM-1 up-regulation as assessed by flow cytometry, whereas NIK inhibition suppressed up-regulation of all three molecules (Fig. 7c, d). In line with this, NIK inhibition prevented LIGHT-induced IL-32, IL-34, CXCL5, and BIRC3 gene expression, whereas p65 inhibition did not affect IL-32 or IL-34, and resulted in only a milder effect on up-regulation of CXCL5 and BIRC3 (Fig. 7e). Considering the early translocation of p65 observed previously, we also investigated the involvement of canonical and non-canonical NF-κB at 4 h post-LIGHT stimulation. LIGHT mediated a moderate increase of ICAM-1 and VCAM-1 protein, as well as IL-32, IL-34 and BIRC3 transcripts after 4 h, at levels significantly below those seen at 24 h (Supplementary Fig. 3). Inhibition of p65 over 4 h did not affect ICAM-1 or VCAM-1 protein, but did block up-regulation of IL-32, IL-34, and BIRC3 transcripts, whereas NIK inhibition again blocked all of the targets tested (Supplementary Fig. 3). In addition, at 24 h, inhibition of p65 also had no effect on LIGHT-driven suppression of transcripts for BMP6, SEMA3B, or other molecules expressed in stem cell niches and found in homeostatic fibroblasts such as the BMP antagonist GREM2^8,33^, and only partially blocked down-regulation of WNT2B (Fig. 7f). In contrast, inhibition of NIK prevented LIGHT-mediated suppression of BMP6, GREM2 and SEMA3B completely, and partially prevented down-regulation of WNT2B (Fig. 7f). Collectively, our data support a dominant role of LIGHT effects via LTβR to activate the non-canonical NF-κB pathway when promoting a pro-inflammatory phenotype in esophageal fibroblasts. Our data further demonstrate a supplemental role for canonical NF-κB signals that are derived from both LTβR and HVEM activation.

## Discussion

Fibroblasts have long been recognized as essential components of the stroma that provide structural integrity to tissues and contribute to healing after damage or injury via the production of proteins such as collagens^34,35^. In response to the pleiotropic cytokine TGF-β1, fibroblasts enhance the expression of matrix proteins including collagens or fibronectin, and modify their cytoskeleton with the induction of genes such as ACTA2 or FLNA^5,17,36,37^. Thus, excessive TGF-β1-mediated responses have been thought to be one of the main mechanisms whereby fibroblasts become pathologically activated and participate in unbridled tissue fibrosis that leads to organ dysfunction. However, evidence from a variety of tissues reveals that fibroblasts also may participate in the maintenance of homeostasis and play important roles in coordinating inflammatory responses^1,2,4,38^. Our studies demonstrate that LIGHT is one molecule that can drive an inflammatory transcriptional and eosinophil-tethering phenotype^17^. Here we build on this and show that LIGHT is a central regulator of fibroblast function in the esophagus by also repressing mediators that are likely to be involved in esophageal epithelial homeostasis. We show that fibroblasts that express these factors are present in the normal esophagus and diminished during EoE, whereas fibroblasts expressing inflammatory molecules are dominant in active disease. We define an essential role of LTβR as the main regulator of the LIGHT-mediated inflammatory transcriptome and show that HVEM augments the activity of LTβR for a subset of the inflammatory targets. Lastly, we show that the NIK-dependent non-canonical NF-κB pathway is the primary signaling cascade downstream of LIGHT that promotes an inflammatory fibroblast phenotype.

Studies of the stromal environment of skin, intestine and joints comparing normal and disease contexts have shown the presence of fibroblasts expressing distinct transcriptional profiles that suggest their participation in previously unrecognized processes^1,4,38,39^. Stromal cell populations were considered a source of WNT factors that contribute to intestinal epithelial organization in exhaustive histologic studies^9^. In line with this, a recent report investigating normal colon samples defined the differential localization of fibroblasts producing epithelial-sustaining factors along the colonic crypt-villi axis. These factors included WNTs, SEMA3, IGFBPs, BMPs and GREMs^8^. Other studies suggested that PDGFRα + pericryptal intestinal stromal cells produce stem cell-stimulating factors such as WNTs and RSPO, and mesenchymal cells expressing R-spondin3, a WNT pathway agonist, were critical for epithelial recovery after bacterial or chemically induced epithelial damage^10,31^. In line with this, the expression of the WNT inhibitor DKK1 contributed to murine colitis that was improved in DKK1 deficient mice^40^. Notably, single cell studies also showed that a colon stromal cell subset expressing epithelial sustaining factors is reduced in patients with ulcerative colitis (UC)^4^. In the context of EoE, where alterations in epithelial permeability as well as basal zone epithelial hyperplasia are common features of disease, the role of WNTs and other epithelial sustaining signals remains under investigation. WNT signals, including WNT2B and WNT5A, have been found in specific areas of the esophageal mucosa and may be involved in the proliferation of esophageal epithelial cells^41^. Similarly, BMPs and GREM1 are produced by esophageal myofibroblasts and contribute to epithelial growth and BMP-mediated responses contribute to basal zone hyperplasia in EoE^42,43^. Adding to this, our study shows that esophageal fibroblasts from different donors express a similar regulatory transcriptional profile to that identified in intestinal tissues, including WNT factors (WNT5A, WNT2B), WNT receptors (FZD4) and targets (OLFM2), insulin growth factor binding proteins (IGFBP3, IGFBP5), semaphorins (SEMA3B and SEMA3D) and bone morphogenetic proteins and antagonists (BMP6 and GREM2). Interestingly, in situ analysis of healthy esophageal specimens and EoE biopsies shows the existence of a population of VIM^+^WNT2B^+^ cells, presumably fibroblasts, that localize in close proximity to the basal epithelium. These cells are lost in active EoE, and fibroblasts isolated from active EoE patients demonstrate down-regulation of WNT2B coupled with an increase in the WNT inhibitor DKK2 in their basal transcriptome, suggesting this is a consequence of inflammation in the esophagus. Importantly, we found that LIGHT is a direct repressor of the transcription of these genes, while it up-regulates the WNT inhibitor DKK1. Thus, adding to our previous observation of the ability of LIGHT to up-regulate inflammatory mediators, this evidence shows that LIGHT suppresses factors that are potentially important in tissue homeostasis. However, further studies are needed to clarify the relevance of some of these molecules and their specific contribution to the maintenance of esophageal epithelial homeostasis.

LIGHT signaling is complex due to its ability to bind to two different receptors (HVEM and LTβR) with high affinity. A significant number of past studies have focused on defining the specific contribution of either receptor to LIGHT activity, with different results seen depending on the tissue or cell type investigated. For example, in studies of human lung fibroblasts, we showed that HVEM is dispensable for many LIGHT-driven responses^20^. In contrast, studies of human keratinocytes showed that HVEM was equally important for promoting proliferation as LTβR. Additionally, HVEM specifically up-regulated the remodeling factor periostin, an activity not induced by LTβR^21^. Here, we show that HVEM expression is different between fibroblasts from inactive and active EoE with more HVEM in EoE fibroblasts consistent with higher HVEM expression in EoE biopsies as compared with normal esophagus. By contrast, LTβR remains stably expressed regardless of disease state. TGF-β1 enhances HVEM expression and this augments the ability of LIGHT-LTβR signals to drive expression of several inflammatory factors, including enhancing ICAM-1 and IL-32 expression, as well as repressing expression of several homeostatic factors such as WNTs, SEMA3B or BMP6. Based on this data, we propose that LIGHT induces an LTβR-dependent inflammatory transcriptome in less differentiated esophageal fibroblasts that is enhanced and/or reinforced by HVEM signaling, whereas fibroblasts exposed to TGF-β1 that differentiate into myofibroblasts will exhibit an exaggerated response to LIGHT that is still LTβR-dependent but more influenced by HVEM due to its increased expression.

Fibroblasts had previously been proposed as interactors with other immune cell types such as T cells and eosinophils^44,45^. In keeping with this, we showed that fibroblasts tether eosinophils in vitro, that high eosinophilia correlates positively with the abundance of ICAM-1+ fibroblasts extracted from active EoE patients and cultured in vitro, and that blockade of ICAM-1 leads to reduced eosinophil adhesion in co-culture with fibroblasts^17^. Adding to this, our current study demonstrates that knock down of either HVEM or LTβR leads to reduced ICAM-1, and that LIGHT-dependent fibroblast-eosinophil tethering is reduced by a silencing of either receptor. These data support the role of the HVEM/LTβR-ICAM-1 axis in fibroblasts-eosinophil tethering. Our previous studies also showed that VIM^+^ and EPX^+^ cells co-localize in EoE biopsies. Adding to this, here we found that populations of VIM^+^ cells expressing either ICAM-1 or IL-34 or both are increased in the LP and EPI compartment of the EoE esophagus. Moreover, we found that fibroblasts isolated from active EoE biopsies display increased expression of several markers of the LIGHT-driven phenotype, and that some of the LIGHT-induced mediators are also up-regulated in biopsies from active EoE patients. Therefore, our past and present results add to the concept of an inflammatory fibroblasts that emerges in disease and contributes to the recruitment of immune cells, showing a key role of LIGHT and its receptors as mediators of these responses in the esophagus.

LTβR is considered one of the main receptors promoting the cleavage of p100 into p52 thereby activating non-canonical NF-κB signaling, although other studies suggest that LTβR can also trigger nuclear translocation of p65^46,47^. In contrast, the role of HVEM in the activation of these responses is not well understood, although prior studies suggest that HVEM may not be a direct driver of p100 cleavage, whereas its role in the activation of p65 has been suggested^48,49^. In our studies, LIGHT activated both p65 nuclear translocation and p100 cleavage with different kinetics. Knock-down of HVEM had no effect on p100 cleavage and moderately blocked p65 translocation, whereas a loss of LTβR strongly suppressed p100 cleavage and also affected p65 translocation. Moreover, we found that inhibiting p65 translocation had little effect on targets such as ICAM-1 or CXCL5, which were down-regulated by siHVEM, whereas inhibition of p100 cleavage with a small molecule NIK inhibitor suppressed inflammatory gene expression in a manner that is consistent with the effects found for siLTβR, at early (4 h) and late (24 h) post-LIGHT stimulation time periods. Thus, the LTβR-NIK-p100/p52 pathway appears to be the dominant pathway mediating LIGHT’s inflammatory gene expression in esophageal fibroblasts. On the other hand, our studies could not fully explain the mechanisms of LIGHT-HVEM-mediated gene expression, since IL-32, which can be down-regulated by siHVEM after 24 h of LIGHT-mediated stimulation, was only affected by inhibition of p65 at 4 h post-LIGHT but not at 24 h. However, IL-32 was diminished by inhibition of NIK-mediated p100 cleavage and our results show that this pathway is not regulated via HVEM on esophageal fibroblast. Therefore, this indicates that signaling pathways other than canonical NF-κB may be activated via HVEM and contribute to LIGHT signaling. Possibilities include activation of STAT3 or MAP kinases, with further studies required to fully elucidate the molecular mechanism of LIGHT/HVEM-induced gene expression^50^.

In summary, our results show that LIGHT mediates a shift in the pattern of gene expression in esophageal fibroblasts toward an inflammatory profile. We show that expression of LIGHT-repressed targets is lost in active EoE biopsies and fibroblasts and LIGHT-induced inflammatory mediators are increased. We identify at least two distinct pathways that are likely important from the two LIGHT-receptors, but with LTβR-dependent non-canonical NF-κB being central to the action of LIGHT in promoting this fibroblast differentiation that is a likely contributor to inflammation in EoE. As such, our studies identify novel targets, including LIGHT, its receptors and downstream non-canonical NF-κB as modulators of fibroblast function with therapeutic potential in EoE.

## Methods

### Reagents

Details on all chemicals, antibodies, primers, siRNAs, constructs and RNAscope probes used in this study can be found as Supplementary Material.

### Fibroblast extraction, culture and treatment

Human primary esophageal fibroblasts were obtained from esophageal mucosa of healthy donors (Arkansas Regional Organ Recovery Agency) or from esophageal biopsies of patients with active and inactive EoE (University of California San Diego/Rady Children’s Hospital cohort of EoE subjects). Cells from 14 different healthy donors and 12 EoE patients were used in this study. Details on patients included in this study can be found in the supplementary material section. Active EoE was defined as patients that presented with higher than 15 eosinophils in a high power field (HPF) in an esophageal biopsy, whereas inactive patients were EoE patients under therapy that presented with less than 15 eosinophils per HPF. Cells were cultured in SMCM supplemented with 2% serum, SMCGS and 100 units/ml penicillin and 100 μg/ml streptomycin. For experiments, fibroblasts between passages 2–5 were used and switched to basal SMCM containing 100 units/ml penicillin and 100 μg/ml streptomycin 16–24 h prior to treatment. Cells were exposed to 50 ng/ml LIGHT for 24 h unless otherwise stated. Chemical inhibitors BAY11-7082 (1 µM) and NIK-SMI1 (10 µM) were added 1 h prior to cytokine treatment.

### Transfection

Cells were plated on 6 well plates, grown to 70% confluence and serum deprived for 8–16 h. For transfection siNTC or siHVEM or siLTR or pCMV6-XL4 or pCMV6-XL4-HVEM were pre-diluted in optimem (mix A), HiPerct diluted in optimem separately (mix B) and both pre-incubated for 5 min. Mixes A and B were then combined (transfection mix) and incubated for 20 min. 1 ml of medium was removed from wells and replaced with transfection mix to a 1:1 ratio. Cells were transfected for 24 h prior to cytokine treatment and maintained in medium:transfection mix 1:1 for the duration of the experiment.

### Co-culture studies

eosinophils were extracted from whole blood of human donors, pre-treated with 10ng/ml IL-5 for 48 h and re-suspended in SMCM basal. Fibroblasts were transfected with siNTC or siHVEM or siLTβR and treated with LIGHT as indicated. Medium containing transfection mix or LIGHT was removed from fibroblast monolayers and replaced with medium containing eosinophils added at a 1:1 fibroblast-eosinophil ratio. Fibroblast-eosinophil co-cultures were maintained for 8 h, fixed and stained according to our previously developed method^17^.

### Analysis of RNA sequencing data from human esophageal biopsies and EoE fibroblasts

Publicly available RNA-Seq data were used for esophageal biopsy specimens of patients with EoE or control subjects (GSE58640)^30^. The data were stored in different formats such as read count and TPM. This hinders the integrative analysis of the RNA-Seq data. To ensure fair comparisons across different datasets, we converted RNA-Seq count data (GSE58640) to transcripts per million (TPM). For RNA-Seq analysis of active EoE fibroblasts, passage matched normal and active EoE fibroblasts were cultured to confluence in DMEM supplemented with 10% heat inactivated fetal bovine serum. The raw data is available from the corresponding author upon reasonable request. For comparison of the transcriptome of LIGHT-treated fibroblasts, we used our previously published data set (GSE143482). Quantification and statistical analysis were performed according to previously published methods^17^.

### RNA in situ hybridization

RNAscope (ACDBio) was performed according to manufacturer’s protocols. Histology specimens used in this study were formalin fixed and paraffin embedded. Active EoE specimens were obtained from pediatric EoE patients, defined as patients showing higher than 15 eosinophils in a HPF. Details on patients included in this study can be found in the supplementary materials section.

### Statistical analysis

One-way ANOVA analysis of variance in the group means was performed for multiple comparisons using Newman-Keuls post-test. For experiments comparing only 2 groups, two-tailed unpaired Student’s T-Test with a confidence level of 95% was used. Standard deviation is used in this study. Data was considered significant if the *p* value was < 0.05 (*), or < 0.01 (**) or 0.001 (***).

## Supplementary information

Supplementary materials
