# Supplementary material for: LIGHT controls distinct homeostatic and inflammatory gene expression profiles in esophageal fibroblasts via differential HVEM and LTβR-mediated mechanisms
Source: Mucosal Immunol. 2021 Dec 13;15(2):327–37. doi: 10.1038/s41385-021-00472-w (PMC8866113; doi:10.1038/s41385-021-00472-w)
Supplement: Supplementary file 1 — Supplementary materials [file 41385_2021_472_MOESM1_ESM.pdf]

Supplementary materials

Reagents

Reagent	brand	code	Application
**Recombinant human TNFSF14/LIGHT**	R&D systems	#664-LI-025/CF	Cell stimulation
**NIK-SMI1**	ProbeChem	PC-62514	Cell treatment
**BAY11-7082**	Cayman chem	10010266	Cell treatment
**HiPerfect**	Qiagen	301705	Cell transfection
**Smooth muscle cell medium (SMCM)**	ScienCell	1101-b	Cell culture
**Smooth muscle cell growth supplements (SMCGS)**	ScienCell	1152	Cell culture
**Dulbecco's modified essential medium**	Gibco	11995-065	Cell culture
**Opti-MEM® I**	Gibco	31985-070	Transfection
**Penicillin/Streptomycin**	ScienCell	0503	Cell culture
**Foetal Bovine Serum**	ScienCell	0010	Cell culture

Short interfering RNAs and plasmids

Reagent	Brand	Code	Dose used
**siNTC**	Dharmacon	D-001810-10-05	40nM
**siHVEM-B**	Origene	SR305773B	40nM
**siLTβR**	Dharmacon	L-008023-00-0005	25nM
**pCMV6-XL4-HVEM**	Origene	SC117733	20ng/ml
**pCMV6-XL4**	Origene	pCMV6-XL4	20ng/ml

Patient details

Patient	Gender	Age	Eosinophil count d/m/p	Use
**1**	M	13	40/70/>100	Fibroblasts
**2**	M	10	8/0/0	Fibroblasts
**3**	M	15	>100/75/6	Fibroblasts
**4**	M	12	0/0/0	Fibroblasts
**5**	M	5	3/0/0	Fibroblasts
**6**	F	2	0/0/0	Fibroblasts
**7**	F	19	100/30/45	Fibroblasts
**8**	M	16	100/100/72	Fibroblasts
**9**	Unknown	9	1/2/1	Fibroblasts
**10**	F	11	3/0/0	Fibroblasts
**11**	M	12	10/100/100	Fibroblasts
**12**	M	18	30/55/20	Fibroblasts/histology
**13**	M	8	27/2/0	Histology
**14**	F	9	30/0/0	Histology
**15**	M	7	85/10/85	Histology
**16**	M	10	>100/>100/>100	Histology
**17**	M	4	22/23/44	Histology
**18**	M	11	44/53/60	Histology
**19**	M	10	60/1/0	Histology

Primers

Gene	Primer	5’-3’
**RPL13A**	FWD	CATAGGAAGCTGGGAGCAAG
	REV	GCCCTCCAATCAGTCTTCTG
**ICAM1**	FWD	TTGTTGGGCATAGAGACCCC
	REV	GGTTTTAGCTGTTGACTGCCC
**IL32**	FWD	AGAGCTGGAGGACGACTTCA
	REV	CTCGGCACCGTAATCCATCT

**IL33**	FWD	CATCTGGTACTCGCTGCCTGTC
	REV	CAACACCGTCACCTGATTCATT
**IL34**	FWD	CTTGGGATCTTCCTTGGCGT
	REV	CAGCTTGTCCCGCAGAAAAC
**CXCL5**	FWD	CACGCAAGGAGTTCATCCCA
	REV	TCCTTCCCGTTCTTCAGGGA
**BIRC3**	FWD	TCATCCGTCAAGTTCAAGCCA
	REV	TCTCCTGGGCTGTCTGATGT
**DKK1**	FWD	TGACAACTACCAGCCGTACC
	REV	CGAGACAGATTTGCACGCCT
**WNT2b**	FWD	GATCCGAGAGTGTCAGCACC
	REV	CTGCCTCTCGGCTACTTCTG
**WNT5a**	FWD	TTTGGCAGGGTGATGCAGAT
	REV	GCCATAGTCGATGTTGTCGC
**BMP6**	FWD	TCATTGCACCCAAGGGCTAT
	REV	GCACGGTTTGGGGACATACT
**SEMA3B**	FWD	CCTCCCTCAACCTGGACAAC
	REV	GCAAATGGGTGCGGTTGTAG
**HVEM**	FWD	GACCCTGGAGGAATGTCAGC
	REV	CGAGGCTCCCTGAGAGAAAC
**LTβR**	FWD	CCGACACAACCTGCAAAAAT
	REV	GAGCAGAAAGAAGGCCAGTG

Antibodies

Reagent	brand	code	Application
**Anti-ICAM1-PE**	eBioscience	#12-0549-42	Flow cytometry
**Anti-VCAM1-APC**	Biolegend	305810	Flow cytometry
**Anti-CD74-PerCP**	Biolegend	357607	Flow cytometry
**Anti-HVEM-APC**	Biolegend	318808	Flow cytometry
**Polyclonal goat anti-LTβR**	R&D systems	AF629	Flow cytometry
**Anti-p65**	Cell signaling technology	8242S	Western blot and immunofluorescence
**Anti-p100/p52**	Cell signaling technology	3017T	Western blot
**Anti-GAPDH**	Cell signaling technology	5174S	Western blot
**Anti-Lamin**	Thermo	MA3-1000	Western blot
**Anti-β-Tubulin**	Cell signaling technology	2128S	Western blot
**Anti-Vimentin**	Sigma	V6389	immunofluorescence
**Anti-WNT2b**	Abcam	Ab178418	immunofluorescence
**Donkey anti-goat IgG Alexa Fluor 488**	Life technologies	#A11055	Flow cytometry
**Alexa fluor 488**	Goat anti-rabbit IgG	Life technologies #A11008	ICC-F
**Alexa fluor 488**	Goat anti-mouse	Invitrogene, #A10680	IF-P
**Alexa fluor 594**	Goat Anti-rabbit	Life technologies, #A11012	IF-P
**Isotype**	Goat IgG control	R&D systems, #AB-108-C	Flow cytometry

**Isotype-PE**	Mouse IgG1κ	eBioscience, #12-4714-82	Flow cytometry
**Isotype-APC**	Mouse IgG1	Biolegend, #406609	Flow cytometry
**Isotype**	Mouse IgG	Abcam, #ab190475	IF-P
**Isotype**	Rabbit polyclonal IgG	Abcam, #ab27478	IF-P
**Phalloidin iFluor 594**		ab176757	IF-C

RNAscope probes

**Reagent**	**Brand**	**code**	**Dilution**
**Hs-vimentin**	ACD-Biotechne	310441-C2	1:50
**Hs-ICAM1**	ACD-Biotechne	402951	Undiluted
**Hs-IL-34**	ACD-Biotechne	313011-C3	1:50
**Hs-WNT2B**	ACD-Biotechne	453361	Undiluted

Supplementary figure 1

A **Diseases and functions**

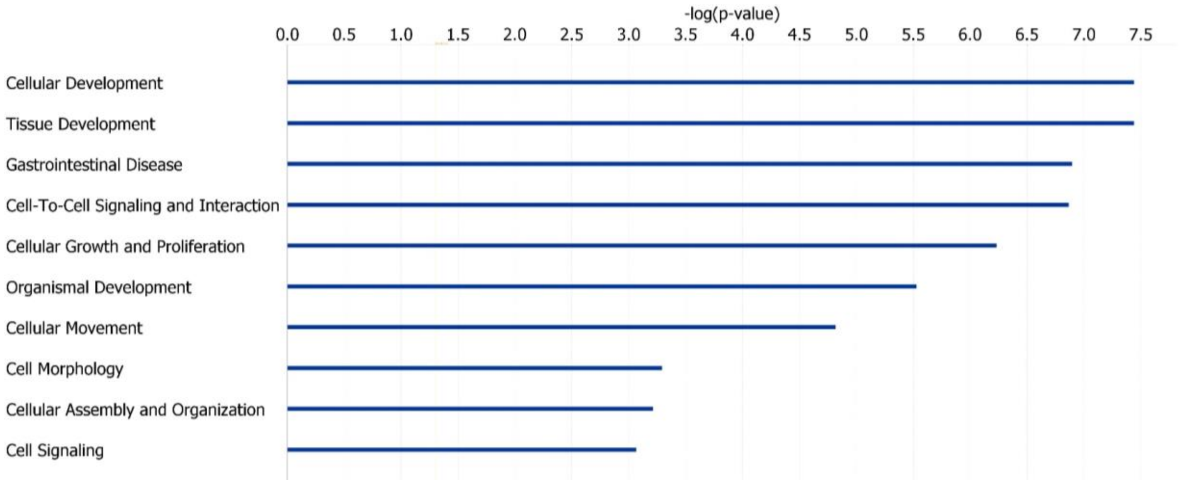

B **Signaling pathways**

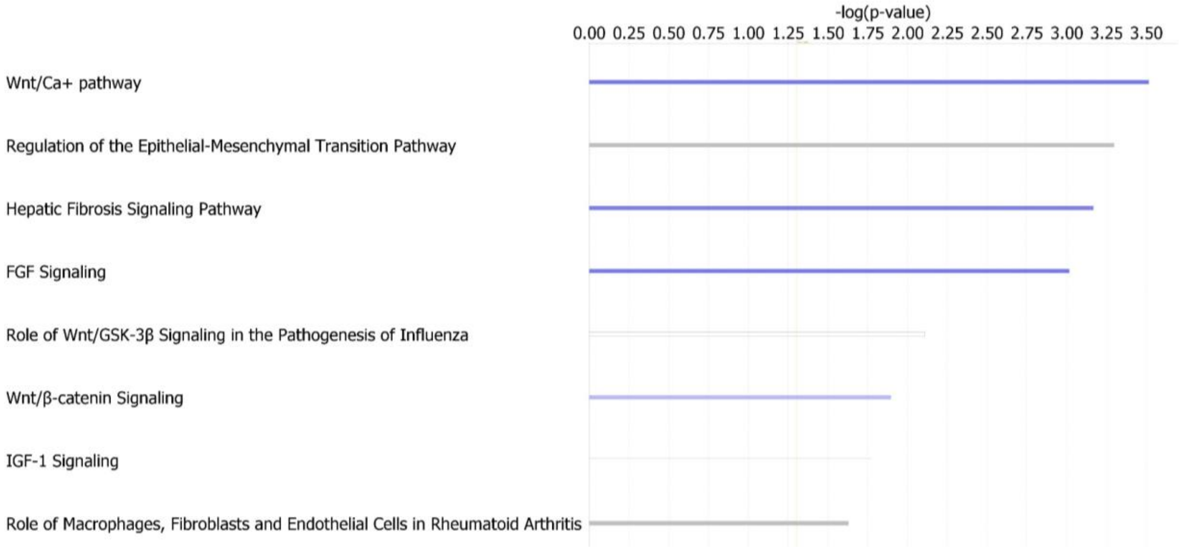

Supplementary figure 2

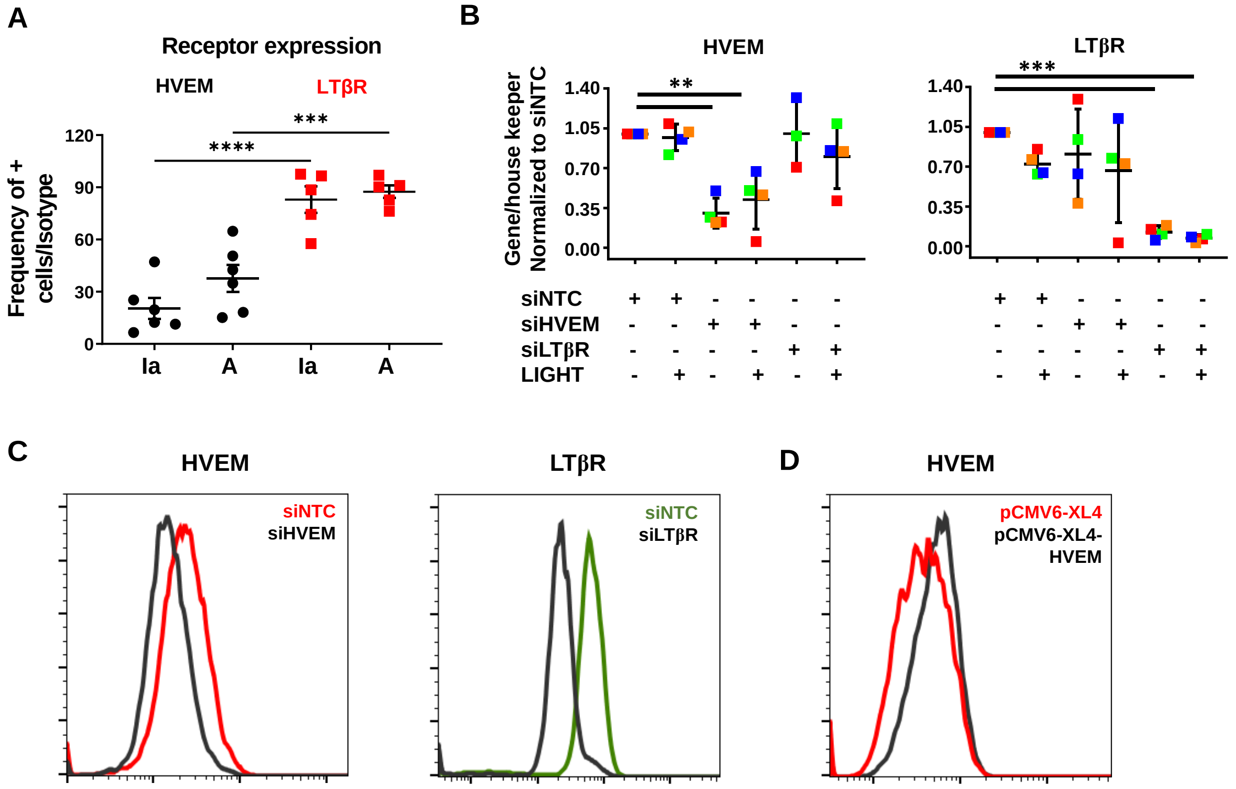

Supplementary figure 3

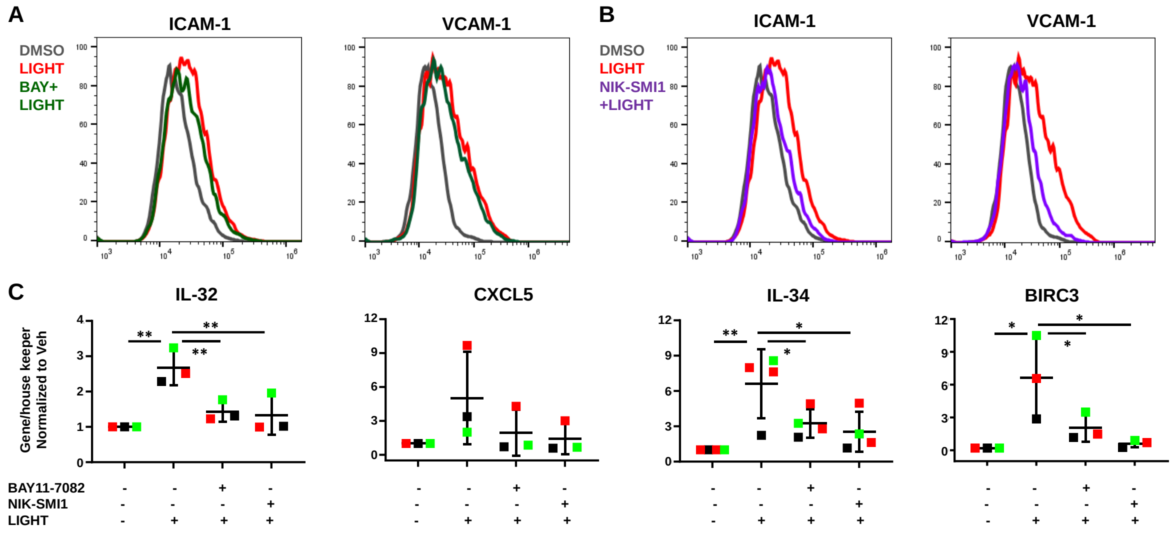

Supplementary Figure 1. Ingenuity Pathway Analysis of LIGHT down-regulated genes showing relevant diseases and functions and signaling pathways (n=4, >1.5 fold, p<0.05).

Supplementary figure 2. A, quantification of % of HVEM and LTβR positive cells normalized to isotype from plots shown in Figure 1C (n=6, *p<0.05). Validation of HVEM and LTβR silencing in cells transfected with scramble RNA (siNTC) or siRNAs against HVEM (siHVEM) or LTβR (siLTβR) and untreated or treated with LIGHT for 24 hours analyzed by RT-PCR (B, n=4), or in cells transfected with siNTC and siHVEM or siLTβR (C, n=3) or with pCMV6-XL4 or pCMV6-XL4L-HVEM analyzed by flow cytometry (D, n=3). Each colored dot represents fibroblasts from an independent donor. *p<0.05 and ***p<0.001.

Supplementary figure 3. Representative histograms of flow cytometry of ICAM-1 and VCAM-1 in normal esophageal fibroblasts pre-treated with BAY11-7082 (A) or NIK-SMI1 (B) for 1 h and then treated with LIGHT for 4h (n=3). RT-PCR of inflammatory genes in esophageal fibroblasts pre-treated with BAY11-7082 or NIK-SMI1 for 1h and then treated with LIGHT for 4h (C, n≥3). Each color dot represents fibroblasts from an independent donor. *p<0.05, **p<0.01.
